# Lithocholic Acid Is a Vitamin D Receptor Ligand That Acts Preferentially in the Ileum

**DOI:** 10.3390/ijms19071975

**Published:** 2018-07-06

**Authors:** Michiyasu Ishizawa, Daisuke Akagi, Makoto Makishima

**Affiliations:** Division of Biochemistry, Department of Biomedical Sciences, Nihon University School of Medicine, 30-1 Oyaguchi-kamicho, Itabashi-ku, Tokyo 173-8610, Japan; ishizawa.michiyasu@nihon-u.ac.jp (M.I.); akagi@risu-s.com (D.A.)

**Keywords:** vitamin D receptor, vitamin D, lithocholic acid, bile acid, CYP24A1, TRPV6, calcium metabolism, ileum

## Abstract

The vitamin D receptor (VDR) is a nuclear receptor that mediates the biological action of the active form of vitamin D, 1α,25-dihydroxyvitamin D_3_ [1,25(OH)_2_D_3_], and regulates calcium and bone metabolism. Lithocholic acid (LCA), which is a secondary bile acid produced by intestinal bacteria, acts as an additional physiological VDR ligand. Despite recent progress, however, the physiological function of the LCA−VDR axis remains unclear. In this study, in order to elucidate the differences in VDR action induced by 1,25(OH)_2_D_3_ and LCA, we compared their effect on the VDR target gene induction in the intestine of mice. While the oral administration of 1,25(OH)_2_D_3_ induced the *Cyp24a1* expression effectively in the duodenum and jejunum, the LCA increased target gene expression in the ileum as effectively as 1,25(OH)_2_D_3_. 1,25(OH)_2_D_3_, but not LCA, increased the expression of the calcium transporter gene *Trpv6* in the upper intestine, and increased the plasma calcium levels. Although LCA could induce an ileal *Cyp24a1* expression as well as 1,25(OH)_2_D_3_, the oral LCA administration was not effective in the VDR target gene induction in the kidney. No effect of LCA on the ileal *Cyp24a1* expression was observed in the VDR-null mice. Thus, the results indicate that LCA is a selective VDR ligand acting in the lower intestine, particularly the ileum. LCA may be a signaling molecule, which links intestinal bacteria and host VDR function.

## 1. Introduction

The vitamin D receptor (VDR) mediates the physiological functions of the active form of vitamin D, 1α,25-dihydroxyvitamin D_3_ [1,25(OH)_2_D_3_], including calcium and bone metabolism, immunity, and cardiovascular function [1,2]. 1,25(OH)_2_D_3_ and its synthetic derivatives also exhibit many pharmacological effects, including the regulation of cellular proliferation and differentiation and lipid metabolism, through VDR activation [3]. VDR undergoes a ligand-dependent conformational change that results in a dynamic interaction with the heterodimer partner retinoid X receptor (RXR) and an exchange of cofactor complexes [4]. The RXR−VDR heterodimer binds preferentially to a specific DNA element that consists of a two-hexanucleotide (AGGTCA or a related sequence) direct repeat motif separated by three nucleotides, known as a direct repeat 3 element, in target genes, such as cytochrome P450 (CYP) 24A1 (gene symbol, *CYP24A1*), and the transient receptor potential vanilloid (TRPV) type 6 (gene symbol, *TRPV6*). CYP24A1 catabolizes 25-hydroxyvitamin D_3_ and 1,25(OH)_2_D_3_ into biological inactive metabolites through 24-hydroxylatoin, a negative feedback mechanism in vitamin D signaling [2,4]. VDR is also activated by bile acids, such as lithocholic acid (LCA) and its metabolite, 3-ketocholanic acid. The VDR activation by these bile acids and 1,25(OH)_2_D_3_ induces the expression of CYP3A enzymes, which metabolize drugs and secondary bile acids in the xenobiotic metabolism pathway [5,6]. Human CYP3A4 can inactivate 1,25(OH)_2_D_3_ [7,8]. LCA treatment also induces the expression of *CYP24A1* in intestinal cells and osteoblasts [9,10]. The VDR activation by LCA may induce vitamin D insufficiency or deficiency by enhancing vitamin D catabolism.

Bile acids are the major metabolic products of cholesterol and are essential for the intestinal digestion and absorption of hydrophobic nutrients, such as triglycerides, fatty acids, cholesterol, and lipid-soluble vitamins, including vitamin D [11]. The primary bile acids, cholic acid and chenodeoxycholic acid, are generated from cholesterol by the sequential action of the liver enzymes and are secreted in the bile as glycine or taurine conjugates [12]. After assisting in the digestion and absorption of lipid compounds, most of the bile acids are reabsorbed in the ileum and enter the enterohepatic circulation. Bile acids that escape reabsorption are converted to the secondary bile acids, deoxycholic acid and LCA, by the intestinal microflora [13]. While deoxycholic acid is avidly accumulated in the enterohepatic circulation pool, a small amount of LCA is absorbed in the ileum, sulfated in the liver, excreted into bile, and then lost in feces [14].

VDR can be activated by 1,25(OH)_2_D_3_, synthetic vitamin D derivatives, LCA, and its derivatives [15,16]. The principal physiological effect of vitamin D is to enhance calcium absorption in the upper intestine [2]. Many synthetic vitamin D derivatives induce hypercalcemia, a potential adverse effect which must be addressed to allow for a broader clinical application [3,17]. Apart from the direct effect of vitamin D absorption, a physical link between bile acids and calcium metabolism has not been demonstrated. The secondary bile acids are produced by the intestinal microflora in the lower intestine, particularly the ileum and colon, sites that are not a location of vitamin D-induced calcium absorption. In this study, we investigated whether 1,25(OH)_2_D_3_ and LCA induce selective VDR activation in the intestine of mice.

## 2. Results

### 2.1. LCA Induces CYP24A1 mRNA Expression in the Ileum but Not in the Duodemum or Jejunum

**Figure 1:**
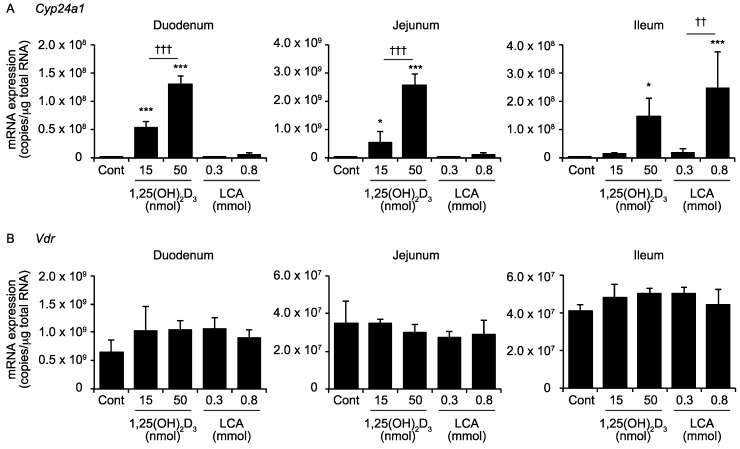
mRNA expression of cytochrome P450 24A1 (*Cyp24a1*) (**A**) and the vitamin D receptor (*Vdr)* (**B**) in the intestine. Wild-type mice were administered the vehicle control (Cont), 15 or 50 nmol/kg 1,25(OH)_2_D_3_, or 0.3 or 0.8 mmol/kg LCA via gavage. * *p* < 0.05; *** *p* < 0.001 versus Cont. †† *p* < 0.01. ††† *p* < 0.001.

*CYP24A1* encodes vitamin D 24-hydroxylase and is the VDR target that is induced by VDR activation in many of the VDR-expressing cells [16]. We treated mice with 1,25(OH)_2_D_3_ (15 or 50 nmol/kg) or LCA (0.3 or 0.8 mmol/kg) by oral gavage twice (14 and 2 h before euthanization), according to our previous reports, with minor modification [9,16,18], and compared the effect of these compounds on *CYP24A1* mRNA expression in the duodenum, jejunum, and ileum. The 1,25(OH)_2_D_3_ treatment increased the *Cyp24a1* mRNA levels in the duodenum, jejunum, and ileum (Figure 1A). LCA was not effective in the duodenum or jejunum, but increased the *Cyp24a1* mRNA levels as effectively as 1,25(OH)_2_D_3_ in the ileum. The 1,25(OH)_2_D_3_ treatment did not change the *Vdr* mRNA levels in the duodenum, jejunum, or ileum (Figure 1B). These findings suggest that LCA is selectively active in the ileum.

### 2.2. LCA Does Not Induce Intestinal Trpv6 Expression or Increase Plasma Calcium Levels

**Figure 2:**
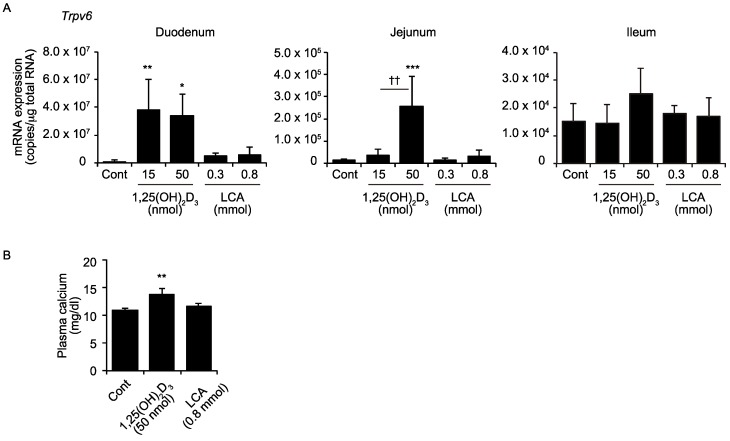
mRNA expression of transient receptor potential vanilloid type 6 (*Trpv6*) in the intestine (**A**) and the plasma calcium levels (**B**). Wild-type mice were administered the vehicle control (Cont), 15 or 50 nmol/kg 1,25(OH)_2_D_3_, or 0.3 or 0.8 mmol/kg LCA via gavage. * *p* < 0.05; ** *p* < 0.01; *** *p* < 0.001 versus Cont. †† *p* < 0.01.

1,25(OH)_2_D_3_ exhibits its principal physiological action of calcium absorption by inducing the calcium channel *Trpv6* in the duodenum [19]. We examined the effect of the oral LCA administration on the *Trpv6* expression, in comparison to 1,25(OH)_2_D_3_. While the 1,25(OH)_2_D_3_ increased the *Trpv6* expression in the duodenum and jejunum, but not the ileum, the LCA did not affect the *Trpv6* expression in the duodenum, jejunum, or ileum (Figure 2). The LCA at 0.8 nmol/kg, which induced ileal *Cyp24a1* expression to similar levels as 1,25(OH)_2_D_3_ at 50 nmol/kg, did not increase plasma calcium levels. The 1,25(OH)_2_D_3_ at 50 nmol/kg increased the calcium levels, consistent with the *Trpv6* expression.

### 2.3. Effects of 1,25(OH)_2_D_3_ And LCA on Expression of VDR Target Genes in the Kidney

**Figure 3:**
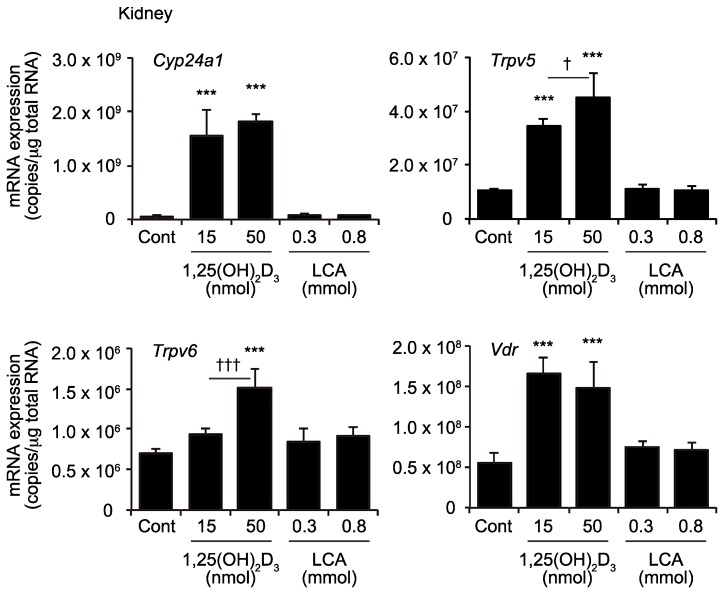
mRNA expression of *Cyp24a1*, *Trpv5*, *Trpv6*, and *Vdr* mRNA in the kidney. Wild-type mice were administered the vehicle control (Cont), 15 or 50 nmol/kg 1,25(OH)_2_D_3_, or 0.3 or 0.8 mmol/kg LCA via gavage. *** *p* < 0.001 versus Cont. † *p* < 0.05; ††† *p* < 0.001.

The kidney is also an important VDR target organ [20], and vitamin D signaling plays a role in calcium reabsorption by the renal tubule through the induced expression of *Trpv5* and *Trpv6* [21]. While oral 1,25(OH)_2_D_3_ treatment induced expressions of *Cyp24a1*, *Trpv*5, *Trpv6*, and in the kidney as reported previously [16,22], LCA was not effective in induction of these genes (Figure 3).

### 2.4. Ileal Cyp24a1 Induction by LCA Is Mediated by VDR Activation

**Figure 4:**
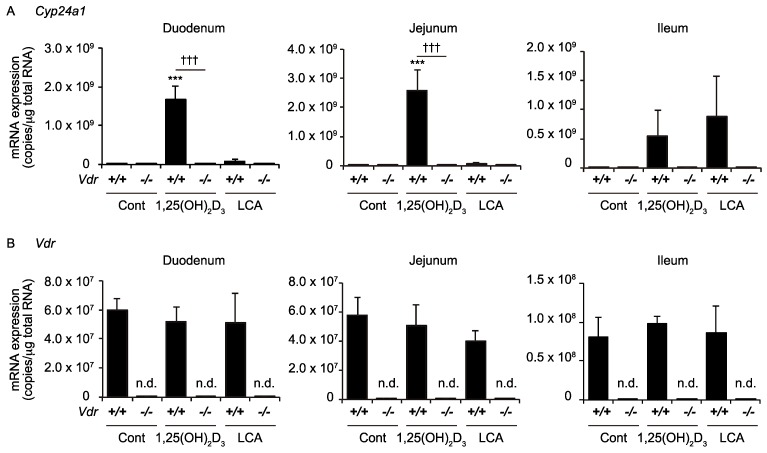
mRNA expression of *Cyp24a1* (**A**) and *Vdr* (**B**) in the intestine of *Vdr(+/+)* mice and *Vdr(−/−)* mice. Mice were administered vehicle control (Cont), 50 nmol/kg 1,25(OH)_2_D_3_, or 0.8 mmol/kg LCA via gavage. *** *p* < 0.001 versus Cont. ††† *p* < 0.001. n.d., not detected.

**Figure A1:**
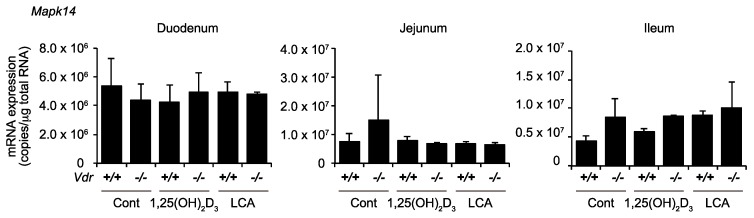
mRNA expression of *Mapk14* in the intestine of *Vdr (+/+)* mice and *Vdr (−/−)* mice. Mice were administered vehicle control (Cont), 50 nmol/kg 1,25(OH)_2_D_3_, or 0.8 mmol/kg LCA via gavage.

LCA is also a ligand for other nuclear receptors, particularly the farnesoid X receptor and pregnane X receptor [6,23,24]. We examined whether the effect of LCA on the ileal *Cyp24a1* induction is mediated by VDR activation utilizing VDR knockout mice [25]. The effect of LCA on the ileal *Cyp24a1* induction was abolished in the *Vdr(−/−)* mice, similar to the effect of 1,25(OH)_2_D_3_, although the effect of 1,25(OH)_2_D_3_ and LCA on the ileal *Cyp24a1* expression did not reach statistical significance because of a large variation (Figure 4A). The *Vdr* mRNA expression was not detected in the *Vdr(−/−)* mice (Figure 4B). The *Vdr(−/−)* mice were generated by targeting exon 2 of the *Vdr* gene and showed no VDR protein expression [25]. The *Cyp24a1* and *Vdr* mRNA levels in the wild-type mice (Figure 4) were different from those shown in Figure 1. This may be due to a difference in the diet conditions. The mitogen activated protein kinase p38α is involved in the intestinal VDR signaling [26]. There was no difference in the expression of *Mapk14*, which encodes p38α, among the experimental groups (Figure A1). We performed a microarray analysis to compare the RNAs purified from the ileum of the LCA-treated *Vdr(+/+)* and *Vdr(−/−)* mice, but found no difference in the expression of the *Rxra*, *Rxrb*, *Rxrg*, or lipid metabolism genes, including *Srebf1* and *Srebf2*.

## 3. Discussion

The secondary bile acid LCA acts as a VDR ligand, is produced by intestinal microflora, and is present mainly in the lower intestine [5,13]. These properties of LCA are different from those of 1,25(OH)_2_D_3_, the essential signaling molecule in the maintenance of calcium homeostasis. The principal site for the 1,25(OH)_2_D_3_ action in calcium absorption is the upper intestine, particularly the duodenum [27]. Consistent with their biological characteristics, our results show that 1,25(OH)_2_D_3_ and LCA induce the VDR target gene *Cyp24a1* preferentially in the upper intestine and lower intestine, respectively (Figure 1).

Dietary vitamin D is absorbed via passive diffusion and through cholesterol transporters, such as Niemann-Pick C1-like 1, scavenger receptor class B type 1, and cluster determinant 36, in the upper intestine [28,29]. Vitamin D is hydroxylated at the 25-position in the liver and then at the 1α-position, to yield the 1,25-dihydroxylated active form in the kidney [2]. 1,25(OH)_2_D_3_, whether it is generated endogenously or taken by oral administration, induces the expression of genes involved in calcium import, such as *Trpv6*, to enhance calcium absorption in the duodenum. 1,25(OH)_2_D_3_ also induces the *Cyp24a1* expression in the upper intestine, and is then inactivated by CYP24A1, leading to decreased activity in the lower intestine. The vitamin D prodrug 1,25(OH)_2_D_3_-25β-glucuronide, which cannot activate VDR or be absorbed in the upper intestine, is converted to free 1,25(OH)_2_D_3_ by intestinal bacteria in the lower intestine [30]. This compound, similar to LCA, exhibits a *Cyp24a1* expression selectively in the lower intestine. LCA is generated by intestinal bacteria and has little reabsorption in the enterohepatic circulation [13]. The orally administered LCA did not induce the expression of the VDR target genes in the kidney (Figure 3), likely because only a small amount of LCA reaches the circulation. The dietary supplementation of LCA induces little change in the blood bile acid composition [31]. Unlike vitamin D, LCA is suggested to be biologically inactive in the upper intestine and to play a role selectively in the lower intestine.

TRPV6 is necessary to mediate the vitamin D-stimulated calcium absorption [19]. In contrast to 1,25(OH)_2_D_3_, LCA did not increase the intestinal *Trpv6* expression or plasma calcium levels (Figure 2). These findings are consistent with an absence of a physiological link connecting the LCA and calcium metabolism. The LCA administration elevates the serum calcium levels and induces renal *Cyp24a1* expression, only in vitamin D-deficient rats [32]. Thus, LCA has only a limited function as a VDR ligand in the body. LCA effectively induces *Cyp24a1* in the ileum (Figure 1). LCA activates other nuclear receptors, farnesoid X receptor and pregnane X receptor, as well as the G protein-coupled receptor, TGR5 [6,23,24,33,34]. The induction of *Cyp24a1* by LCA was not observed in the VDR-null mice (Figure 4), indicating that VDR, but not the other receptors, mediates the effect of LCA on *Cyp24a1* induction.

1,25(OH)_2_D_3_ is also inactivated by CYP3A4, another VDR target gene product, in human cells [8]. LCA induces the *CYP3A4* promoter activity in a VDR-dependent manner in human intestinal LS174T cells [35]. The LCA accumulation may induce vitamin D deficiency or insufficiency by enhancing vitamin D inactivation. The VDR deletion induces a change in the intestinal microbial flora [36], suggesting an interaction between the intestinal microflora and the intestine through the VDR activation by LCA. VDR plays a role in innate immunity and protection against antimicrobial infection [37]. LCA-producing bacteria may induce a favorable environment by regulating host immunity though VDR activation. The VDR regulates the intestinal proliferation, barrier function, and immunity, and plays a protective role in inflammatory bowel diseases [38]. We suggest two possible roles of the LCA−VDR axis. First, LCA induces vitamin D-mimic effects to maintain intestinal homeostasis, a beneficial effect. Second, the LCA decreases the vitamin D signaling by inducing vitamin D catabolism, a pathological effect. Vitamin D deficiency and insufficiency cause rickets and osteomalacia and are also associated with an increased risk of osteoporosis, cancer, autoimmune disease, infection, cardiovascular disease, obesity, and diabetes [1]. Decreased vitamin D signaling induces the differentiation of mesenchymal stem cells into adipocytes not osteoblasts [39,40]. The gene silencing experiments of the VDR and RXR in the intestinal cells and culture experiments using primary cells from *Vdr(−/−)* mice will clarify the LCA−VDR axis.

VDR regulates the target gene expression by forming a heterodimer with RXR [4]. Although the VDR–RXR heterodimer is not permissive to RXR ligand activation, heterodimer allosteric communication is required for activation of VDR by LCA and not by 1,25(OH)_2_D_3_ [41]. LCA induces the interaction of VDR with RXR and cofactors, such as the steroid receptor coactivator 1, silencing mediator of retinoic acid and thyroid hormone receptor, and Hairless, in a manner distinct from 1,25(OH)_2_D_3_ [42,43,44]. The vitamin A derivative, 9-*cis* retinoic acid (9-*cis* RA), has been identified as a natural RXR ligand [45], but it can be detected only in the pancreas [46]. Although fatty acids, such as docosahexaenoic acid and the long chain fatty acid C24:5, have been shown as natural RXR ligands [47,48], their role in intestinal VDR signaling remains unknown. The permissiveness of RXR in the VDR–RXR heterodimer has been investigated using 9-*cis* RA or synthetic ligands, and there may be a natural ligand that can exhibit a permissive or conditionally permissive RXR activation in VDR–RXR. The lower intestine-selective cofactor(s) and/or natural RXR ligand(s) may be involved in LCA-mediated VDR signaling.

We used male mice in this study. Sex-related differences have been reported in the phenotypes of the VDR knockout mice in lipid metabolism and resistance to obesity, and in skeletal structures [49,50], in VDR single nucleotide polymorphisms with human immune, as well as intestinal pathology [38], and also in bile acid metabolism and gut microbiota [51]. Further studies are needed to elucidate the physiological and pathological roles of LCA as a VDR ligand in the intestine.

## 4. Materials and Methods

### 4.1. Animal Experiments

The VDR-null (*Vdr(−/−)*) mice and wild-type mice (*Vdr(+/+)*) mice were obtained by breeding *Vdr(+/−)* mice on a pure C57BL/6J background [52]. Original *Vdr(−/−)* mice were kindly provided by Shigeaki Kato and Chugai Pharmaceutical Co. [25]. These mice were raised on a high-calcium and high-lactose diet to normalize the blood calcium levels in the *Vdr(−/−)* mice, and were maintained under a controlled temperature (23 ± 1 °C) and humidity (45–65%), with free access to water and chow as reported previously [22]. The experiments were conducted with male mice at 8–12 weeks of age. The mice were treated with corn oil containing the vehicle control (ethanol; Kanto Chemical Co., Tokyo, Japan), 1,25(OH)_2_D_3_ (15 or 50 nmol/kg; Wako Pure Chemicals, Osaka, Japan), or LCA (0.3 or 0.8 mmol/kg; Nacalai Tesque, Kyoto, Japan) via oral gavage, twice (14 and 2 h before euthanization), as reported previously, with minor modifications [9,16,18]. The plasma, kidney, and intestine samples were collected after euthanization with carbon dioxide. All of the tissue samples were snap-frozen in liquid nitrogen or on dry ice, and sorted until analysis. The plasma calcium concentrations were quantified with Calcium C Testwako (Wako Pure Chemicals, Osaka, Japan) [16]. The experimental protocol adhered to the Guidelines for Animal Experiments of the Nihon University School of Medicine, and was approved by the Ethics Review Committee for Animal Experimentation of the Nihon University School of Medicine (AP10M096, 3 September 2010; AP12M029, 5 October 2012).

### 4.2. Reverse Transcrption and Real-Time Quantitative Polymerase Chain Reaction

The tissues were crushed using a Bessman Tissue Pulverizer (Spectrum Laboratories, Racho Dominguez, CA, USA), and the total RNA extraction was performed using the acid guanidium thiocyanate/phenol/chloroform method [52]. The cDNAs were synthesized using the ImProm-II Reverse Transcription system (Promega, Madison WI, USA), and the real time polymerase chain reaction was performed on the ABI PIRSM 7000 Sequence Detection System (Life Technologies Corporation, Carlsbad, CA, USA) using Power SYBR Green PCR Master Mix (Life Technologies Corporation) and primers reported previously [16,22]. The mRNA copy numbers were calculated with standard curves that were linear over a range of 0.02–200 ng/mL for the corresponding cloned cDNAs inserted into pcDNA3.1 plasmids (Life Technologies Corporation), as reported previously [26].

### 4.3. Statistical Analysis

Data are presented as means ± standard deviation (SD). We performed one-way ANOVA followed by Tukey’s multiple comparisons to assess significant differences.

## Abbreviations

VDR	vitamin D receptor
1,25(OH)_2_D_3_	1α,25-dihydroxyvitamin D_3_
RXR	retinoid X receptor
CYP	cytochrome P450
TPPV	transient receptor potential vanilloid
LCA	lithocholic acid
